# Selective pressure mediated by influenza virus M1_58–66_ epitope-specific CD8^+^*T* cells promotes accumulation of extra-epitopic amino acid substitutions associated with viral resistance to these T cells

**DOI:** 10.1016/j.virusres.2024.199355

**Published:** 2024-03-15

**Authors:** Janina M. Jansen, Robert Meineke, Antonia Molle, Carolien E. van de Sandt, Giulietta Saletti, Guus F. Rimmelzwaan

**Affiliations:** a Research Center for Emerging Infections and Zoonoses, University of Veterinary Medicine, Hannover, Germany; b Department of Microbiology and Immunology, University of Melbourne at the Peter Doherty Institute for Infection and Immunity, Melbourne, Australia

**Keywords:** Influenza virus, T cells, Immune evasion, Mutation, Epitope

## Abstract

Influenza viruses are notorious for their capacity to evade host immunity. Not only can they evade recognition by virus-neutralizing antibodies, there is also evidence that they accumulate mutations in epitopes recognized by virus-specific CD8^+^*T* cells. In addition, we have shown previously that human influenza A viruses were less well recognized than avian influenza viruses by CD8^+^*T* cells directed to the highly conserved, HLA-A*02:01 restricted M1_58–66_ epitope located in the Matrix 1 (M1) protein. Amino acid differences at residues outside the epitope were responsible for the differential recognition, and it was hypothesized that this reflected immune adaptation of human influenza viruses to selective pressure exerted by M1_58–66_-specific CD8^+^*T* cells in the human population. In the present study, we tested this hypothesis and investigated if selective pressure exerted by M1_58–66_ epitope-specific CD8^+^*T* cells could drive mutations at the extra-epitopic residues *in vitro*. To this end, isogenic influenza A viruses with the M1 gene of a human or an avian influenza virus were serially passaged in human lung epithelial A549 cells that transgenically express the HLA-A*02:01 molecule or not, in the presence or absence of M1_58–66_ epitope-specific CD8^+^*T* cells. Especially in the virus with the M1 gene of an avian influenza virus, variants emerged with mutations at the extra-epitopic residues associated with reduced recognition by M1_58–66_-specific T cells as detected by Next Generation Sequencing. Although the emergence of these variants was observed in the absence of selective pressure exerted by M1_58–66_ epitope-specific CD8^+^*T* cells, their proportion was much larger in the presence of this selective pressure.

## Highlights

• Use of a unique co-culture system to study influenza virus evolution in HLA-A*02:01 transgenic A549 cells under selective pressure by cloned human T cells specific for the conserved M1_58–66_ CTL epitope.
• Upon serial co-culture passages, virus evolution was assessed by next generation sequencing using molecularly cloned recombinant viruses containing the M1 gene of an avian or a human influenza A virus.
• Accumulation of previously described extra-epitopic amino acid substitutions associated with reduced recognition by M1_58–66_ –specific CD8+ *T* cells is promoted by selective pressure mediated by these cells.

## 1 Introduction

Influenza viruses are known for their capacity to evade host immunity induced by previous infections. For example, these viruses accumulate amino acid substitutions in proximity to the receptor binding site of the viral hemagglutinin (HA), allowing them to evade recognition by virus-neutralizing antibodies induced in the human population by infections with ancestral viruses and/or vaccination (Rambaut et al., 2008; van de Sandt et al., 2012). Selective pressure by these antibodies drives this antigenic drift, which necessitates updating the composition of influenza vaccines almost annually to ensure that the vaccine strains match the circulating epidemic strains antigenically, and optimal effectiveness of the vaccines (Koel et al., 2013; Smith et al., 2004). In addition, new strains of influenza A virus (IAV) with novel antigenic properties can emerge after the reassortment of gene segments between IAVs of human and animal origin or following zoonotic transmission (Krammer et al., 2018). Virus-neutralizing antibodies induced by infection with seasonal influenza viruses afford little or no protection against these novel strains, increasing their pandemic potential (Kim et al., 2018).

Virus-specific CD4^+^ and CD8^+^ *T* cells contribute to protective immunity by releasing cytokines and recognizing and eliminating virus-infected cells (Jansen et al., 2019). It has been shown that the presence of pre-existing cross-reactive CD8^+^ *T* cells, mainly directed to the conserved nucleoprotein (NP) and the matrix-1 protein (M1) induced after infection with seasonal influenza viruses, limit severity and duration of disease caused by pandemic influenza A viruses (Gras et al., 2010; Sridhar et al., 2013; Hayward et al., 2015).

In addition to evasion from recognition by VN antibodies, there is evidence that IAVs can evade recognition by virus-specific T cells (van de Sandt et al., 2012; Rimmelzwaan et al., 2004a; Woolthuis et al., 2016; Machkovech et al., 2015). For example, a mutation in the cytotoxic CD8^+^ *T* cell (CTL) HLA-B*27:05/NP_383–391_ epitope was identified at an anchor residue resulting in the loss of the epitope (Voeten et al., 2000a). In addition, amino acid substitutions have been found in CD8^+^ *T* cell receptor contact residues of the HLA-B35:01/NP_418–426_ epitope that abrogated recognition by epitope-specific CD8^+^ *T* cells (Boon et al., 2002; Berkhoff et al., 2007). In both cases, these mutations were sufficient to significantly impair the influenza virus-specific CD8^+^ *T* cell response *in vitro* (Berkhoff et al., 2007; Berkhoff et al., 2004).

Although some IAV CTL epitopes display variation, others are conserved, like the HLA-A*02:01 restricted M1_58–66_ epitope. Despite the high prevalence of HLA-A*02:01 in the human population and the immunodominant nature of the epitope, it remained conserved in seasonal IAVs despite strong immune pressure by specific CD8^+^ *T* cells. It was shown that the epitope does not tolerate amino acid substitutions and is under functional constraints (Berkhoff et al., 2005), which is explained by overlap with a nuclear export signal of the M1 protein (Terajima and Ennis, 2012; Cao et al., 2012). Of particular interest, it was recently demonstrated that M1_58–66_-specific CD8^+^ *T* cells displayed reduced lytic activity and delayed activation after stimulation with M1 protein derived from human seasonal IAV compared to stimulation with M1 protein derived from an avian influenza virus (van de Sandt et al., 2016). Also, recombinant influenza virus carrying the M1 gene of a human influenza virus displayed reduced replication than those with an M1 gene of an avian influenza virus in the presence of M1_58–66_-specific CD8^+^ *T* cells (van de Sandt et al., 2018a). It was hypothesized that the human seasonal influenza viruses display signs of immune adaption and that extra-epitopic amino acid residues play a role in this phenomenon. Five of these residues were found to be different between human and avian IAVs (I15V, K27R, K101R, V115I, T121A), affecting antigen processing and presentation of the M1_58–66_ epitope (van de Sandt et al., 2016; van de Sandt et al., 2018a).

In the present study, we hypothesized that CD8^+^ *T* cells directed to the HLA-A*02:01/M1_58–66_ epitope exert selective pressure and drive the accumulation of extra-epitopic amino acid substitutions associated with reduced activation of these T cells in the M1 protein originating from an avian influenza virus. To this end, isogenic influenza viruses with M1 genes from a human or an avian virus were serially passaged in A549 cells that express HLA-A*02:01 or not, in the presence or absence of M1_58–66-_specific CD8^+^ *T* cells. Viral fitness and nucleotide sequences were analyzed of the progeny virus, we showed that influenza virus M1_58–66_ epitope-specific T cells exert selective pressure and promote the prior described accumulation of extra-epitopic amino acid substitutions (van de Sandt et al., 2016; van de Sandt et al., 2018a).

## 2 Materials and methods

### 2.1 Cells

Human lung carcinoma A549 cells were cultivated in Ham's F-12 K (Kaighn's) Medium (Gibco™) supplemented with penicillin/streptomycin P/S), Glutamax (G), vitamins, non-essential amino acids (NEAA), all at 1 % v/v), Thermo Fisher), 10 % (v/v) heat-inactivated Fetal Bovine Serum (FBS; Gibco Thermo Fisher) (H10F). Transgenic A549 cells that express the HLA-A*02:01 gene (A549_tg-A2_) were generated as described previously (Rimmelzwaan et al., 2004b). A549_tg-A2_ cells were cultured in medium supplemented with 1 µg/ml puromycin (Gibco, Fisher Scientific).

One day before each experiment, the cells were seeded at 0.1 × 10^6^/well in a 48well plate (Corning®) without puromycin. Expression of HLA-A*02:01 was confirmed by staining with a FITC-labelled antibody directed to the HLA-A*02 molecule (FITC Mouse Anti-human HLA-A2; BD Pharmingen; 1:50), and analysis was done by flow cytometry (BD FACS Fortessa; FACS Diva software).

Madin-Darby canine kidney (MDCK) cells were cultivated in DMEM (Dulbecco's Modified Eagle Medium; Gibco™) supplemented with P/S, G, NEAA (all at 1 % v/v, Thermo Fisher), 10 % FBS and they were seeded into 96 well plates one day prior to use in infection experiments.

### 2.2 T cell clone

Cloned CD8^+^ *T* lymphocytes specific for the HLA-A*02:01 restricted M1_58–66_ epitope (GILGFVFTL) were generated and propagated as described previously (Voeten et al., 2000a, 2000b). Cryopreserved cells were thawed and washed in RPMI 1640 medium (Gibco™) supplemented with 1 % penicillin/streptomycin, glutamine, vitamins, non-essential amino acids, respectively, 10 % heat-inactivated FBS (R10F) and then resuspended in RPMI 1640 medium (Gibco™) supplemented with 1 % P/S, G, vitamins, NEAA, respectively, 10 % heat-inactivated Human Serum (Corning®) and 2-mercaptoethanol (Gibco Life Technologies Limited) (R10H).

### 2.3 Viruses

Isogenic influenza viruses were made by reverse genetics based on IAV WSN/33 (H1N1) (van de Sandt et al., 2018a). Recombinant viruses carrying the M1 gene derived from a human seasonal IAV A/Netherlands/178/1995 (H3N2) or an avian IAV A/Vietnam/1194/2005 (H5N1) were produced as previously described and designated WSN-M-hH3N2 and WSN-M-aH5N1, respectively (van de Sandt et al., 2018a). The viruses were concentrated and purified by sucrose gradient centrifugation. Infectious virus titers were determined by titration in MDCK cells as previously described (Rimmelzwaan et al., 1998).

### 2.4 Co-culture of A549 cells and M1_58–66_ epitope-specific T cells

A549 and A549/tg-A2 cells were infected with WSN-M-hH3N2 or WSN-M-aH5N1 at MOI 1. Virus preparations were diluted in Ham's F-12 K Medium with P/S and 1.4 µg/ml of Trypsin-TPCK (Sigma Aldrich) and added to the cells after washing with DPBS (Gibco). Following incubation for 1 h at 37 °C and 5 % CO_2_, the virus inoculum was aspirated, and the cells were washed twice with DPBS. Then, cells of the M1_58–66_ epitope-specific T cell clone were added in H10F supplemented with 1.4 µg/ml Trypsin-TPCK, 50 U/ml IL-2 (Proleukin S; Novartis), 7 ng/ml IL-7 (Immuno Tools) and 50 ng/ml IL-15 (premium grade; MiltenyiBiotec). The co-culture was incubated for 40 h at 37 °C, 5 % CO_2_.

After the incubation, the supernatant was transferred into a 1.5 ml Eppendorf tube, and cells were removed by centrifugation at 300x*g* for 10 min at 4 °C. The subsequent blind passages were performed using 250 µl/well of supernatant from the previous passage. The remaining supernatants were stored at −80 °C until further usage. In total, 10 serial passages were carried out.

### 2.5 Virus titration

One day before virus titration, 20.000/well MDCK cells were seeded into 96 well plates. A 1:10 dilution series of each sample was prepared in DMEM (Dulbecco's Modified Eagle Medium; Gibco™) supplemented with P/S, glutamine, non-essential amino acids (all at 1 % v/v, Thermo Fisher), 2 % sodium-bicarbonate and 0.1% BSA and Trypsin-TPCK (1 μg/ml). After washing the cells twice with DPBS, 200μl/well of the dilution series was added. The plates were incubated for 72 h at 37 °C and 5 % CO2 before collecting the supernatant, which was tested for hemagglutinating activity.

### 2.6 Hemagglutination assay

Fifty μl of each sample was transferred into v-bottom plates before adding 50μl/well of 1 % turkey red blood cells (RBC). After 30 min incubation on ice, the assay was visually examined, and the highest dilution in which agglutination occurred was used to calculate the 50 % tissue culture infection dose (TCID50).

### 2.7 Isolation of viral RNA and RT-PCR

Viral RNA was isolated from cell culture supernatants using the QiaAmP Viral RNA kit (Qiagen) following the manufacturer's protocol. The Superscript IV First Strand Kit (Thermo Fisher Scientific) was used for cDNA synthesis, following the manufacturer's protocol using the following primer (5′ AGCAAAAGCAGG). Subsequently, PCR was performed using M1-specific primers (M1_fw 5′ GCAGGTAGATGTTGAAAGATG; and M1_rev 5′ CGCTGCCTGCTCACTTGATC) to generate amplicons spanning the first 600 bp of the M1 gene according to the protocol of the manufacturer of AmpliTaq Gold® DNA Polymerase (ThermoFisher Scientific). The PCR program consisted of 39 cycles of 30 s at 95 °C, 30 s at 45 °C, and 3 min at 72 °C.

### 2.8 Agarose gel electrophoreses and gel extraction

PCR products were loaded on a 1.3% agarose gel, visible amplicons of 600 bp were cut from the gel, and the DNA was extracted using the GeneJet Gel Extraction Kit (Thermo Fisher Scientific). The samples were eluted in 30 µl of elution Buffer and stored at −80 °C until further analysis.

### 2.9 NGS library preparation

NEBNextra Ultra II kit (New England Biolabs) was used for NGS library preparation following the manufacturer's protocol. To 100 ng in 25 µl of the PCR products, 1.5 µl Enzyme Mix and 3.5 µl reaction buffer were added and then placed into the thermocycler (20 °C for 30 min, 65 °C for 30 min, 10 °C until taken). 15 µl ligation master mix, 0.5 µl enhancer, and 1.25 µl adaptor were added to the samples and then incubated for 1 h at 20 °C. 1.5 µl of USER enzyme was added to the ligation mix and then placed in the thermocycler (37 °C for 20 min, 10 °C hold). Afterward, the samples were purified. 50 µl sample were added to 43 µl AmpureBeads. After 4 min of binding at RT, the tubes were put into a magnetic rack and washed twice with 80 % EtOH. The sample was taken off the rack, resuspended in 17 µl low TE buffer, and binding was allowed for 2 min. The tube was placed on the rack again to place the supernatant into a fresh tube. 12.5 µl Ultra II Q5 Master Mix and 5 µl of a unique barcode was added to 7.5 µl of the purified samples and then run in the thermocycler following the manufacturer's protocol. The samples were purified again, using 22.5 µl AmpureBeads/sample and washing 3x with 80 % EtOH. The pellet was resuspended in 25 µl, transferred into a fresh tube, and its concentration was measured using a Qubit (Thermo Fisher). Next Generation Sequencing was performed using the Illumina MiSeq system at the Research Core Unit Genomics (RCUG) of the Hannover Medical School.

### 2.10 NGS analysis using Geneious

Sequencing on the Illumina MiSeq system with a paired-end sequencing strategy was performed to achieve a target depth of at least 1000x coverage per amplicon. The sequencing run generated ∼250 bp reads. Paired-end reads were set using Geneious Prime software (2022.2.1; Dotmatics). Then, low-quality reads were removed, and the sequences were trimmed using the BBDuk, with a quality score cut-off of Q20. After merging the paired reads, the De Novo Assemble feature generated contingents of similar sequences. Contingents were then aligned to the reference sequence of the M segment of A/Netherlands/178/1995 (H3N2) or avian IAV A/Vietnam/1194/2005 (H5N1), and variant calling was performed using the Geneious Prime Software. Variants were filtered based on a minimum cut-off of 0.05 % and a minimum depth of 100x to ensure high-confidence variant calls. Statistical analyses were performed using Geneious Prime Software.

## 3 Results

### 3.1 Serial passage of virus in A549_tg-A2_ cells in the presence of M1_58–66_-specific CD8^+^*T* cells

**Fig. 1:**
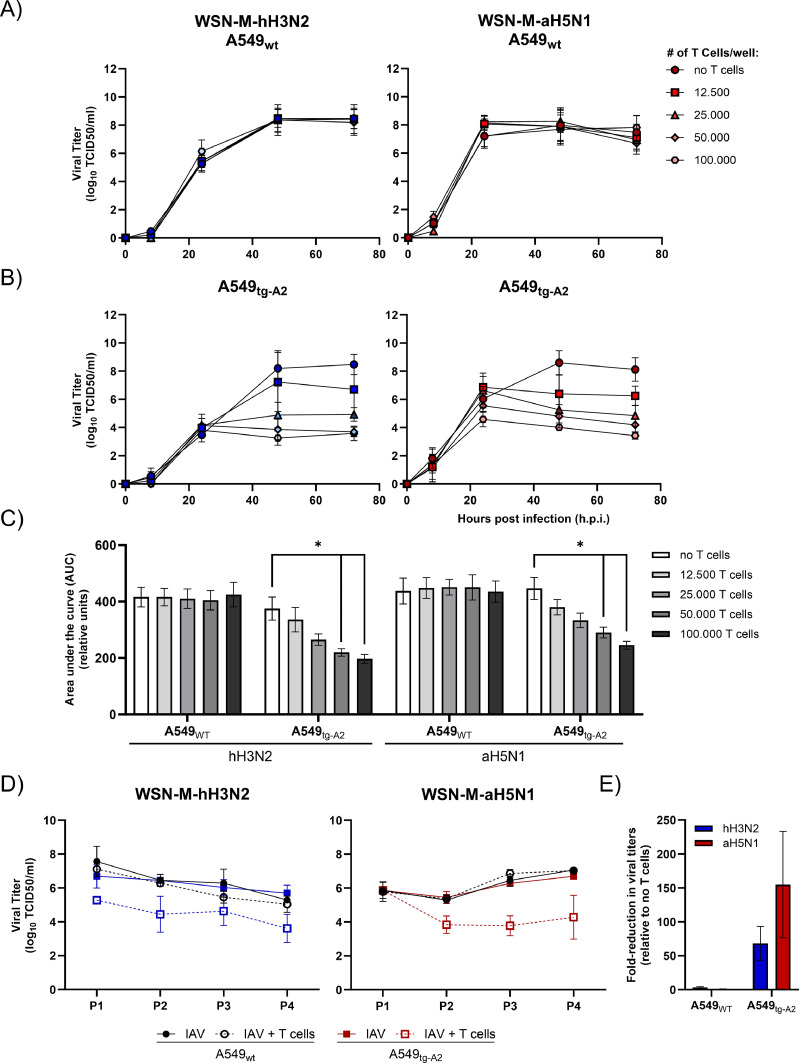
Establishment of efficient virus passaging in A549/ M1_58–66_-specific CTL co-cultures. Virus replication kinetics of WSN-M-hH3N2 and WSN-M-aH5N1 in (A) A549wt and (B) A549tg-A2 cells in the presence of different numbers of M1_58–66_-specific CTL as indicated in the legend. (C) Total virus replication during 72 h of co-cultures. Area under the curve (AUC) is depicted of the grow curves shown in A and B. (D) Viral titers after passaging of WSN-M-hH3N2 and WSN-M-aH5N1 in A549_wt_ (black symbols) and A549_tg-A2_ (blue/red symbols respectively) cells in the presence of 50.000 CTL/well, co-cultured with A549_wt_ cells or A549_tg-A2_ cells expressing HLA-A*02:01. (E) Mean fold-reduction of viral titers in the presence of 50.000 CTL/well, co-cultured with A549_wt_ cells or A549_tg-A2_ cells expressing HLA-A*02:01, relative to the respective wells without CTL (as shown in D). For all data points, median of *n* = 3 (A-C) or *n* = 2 (D-E) and SD are shown.

To assess selective pressure exerted on the M1_58–66_ epitope, a virus-passaging system was established in co-cultures of A549 cells and M1_58–66_-specific CD8^+^ *T* cells, which has been described previously (van de Sandt et al., 2018a). To optimize conditions for serial passaging of the virus, we tested various MOIs of the virus carrying the human M1 gene in A549_wt_ and A549_tg-A2_ cells in the presence of a fixed number of M1_58–66_-specific CD8^+^ *T* cells. Only inoculation of A549 cells at MOI 1 resulted in virus titers high enough for subsequent passaging. In contrast, with MOI 0.01, no infectious virus was detected 24 h post-infection (data not shown). Next, the optimal number of M1_58–66_-specific T cells was determined for serial passaging of the virus. Both A549_wt_- and A549_tg-A2_ cells were infected with the IAVs with the M1 gene of a human (WSN-M-hH3N2) or an avian (WSN-M-aH5N1) IAV. As expected, no differences in virus production could be detected using A549_wt_ cells in the presence or absence of M1_58–66_-specific T cells since the M1_58–66_ peptide cannot be presented by these cells (Fig. 1A and 1C). Virus WSN-M-aH5N1 replicated faster during the first 24 h than WSN-M-hH3N2, but both reached similar virus titers 48 h post-infection. However, in A549_tg-A2_ cells, a reduction in virus titers was observed with increasing numbers of M1_58–66_-specific T cells (Fig. 1B and 1C). For example, a 10,000-fold reduction was observed with 100,000 M1_58–66_-specific T cells per well 72 h post-infection (Fig. 1B). With 50,000 T cells per well, virus titers between 10^3.5^ and 10^4.5^ TCID_50_/ml were observed 48 h post-infection. Additionally, this number resulted in a significant reduction of viral replication (Fig. 1C) and was selected for subsequent passaging experiments. Forty-eight hours post infection, replication of virus WSN-M-hH3N2 with a M gene segment of a human IAV was 6.7-fold higher in the presence of 12,500 M1_58–66_-specific T cells, compared to replication of virus WSN-M-aH5N1 with an M gene segment of an avian IAV. These data confirms that the virus with the M1 gene of a human seasonal influenza virus shows signs of immune adaptation and is more resistant to the action of M1_58–66_ -specific T cells, as was demonstrated previously (van de Sandt et al., 2016; van de Sandt et al., 2018a) (Fig. 1B).

Using the optimized conditions, WSN-M-aH5N1 and WSN-M-hH3N2 viruses were serially passaged ten times in A549_wt_ and A549_tg-A2_ cells. The resulting viral titers were determined for the first four passages. In the absence of HLA-A*02:01 expression, the presence of M1_58–66_-specific T cells did not affect the production of both viruses (Fig. 1D). In contrast, passaging of the viruses in the presence of 50.000 M1_58–66_-specific T cells in A549_tg-A2_ cells resulted in 150- and 60-fold mean reduction in virus replication during four passages of WSN-M-aH5N1 and WSN-M-hH3N2, respectively (Fig. 1E). This again reflects the relative resistance of WSN-M-hH3N2 to the action of M1_58–66_-specific T cells. The virus titers of WSN-M-aH5N1 and WSN-M-hH3N2 remained at the same level during subsequent passages, and we passaged the viruses further up to passage 10. Culture supernatants were collected, and selected samples were submitted for NGS analysis.

### 3.2 Next generation sequencing to screen for adaptive mutations

**Fig. 2:**
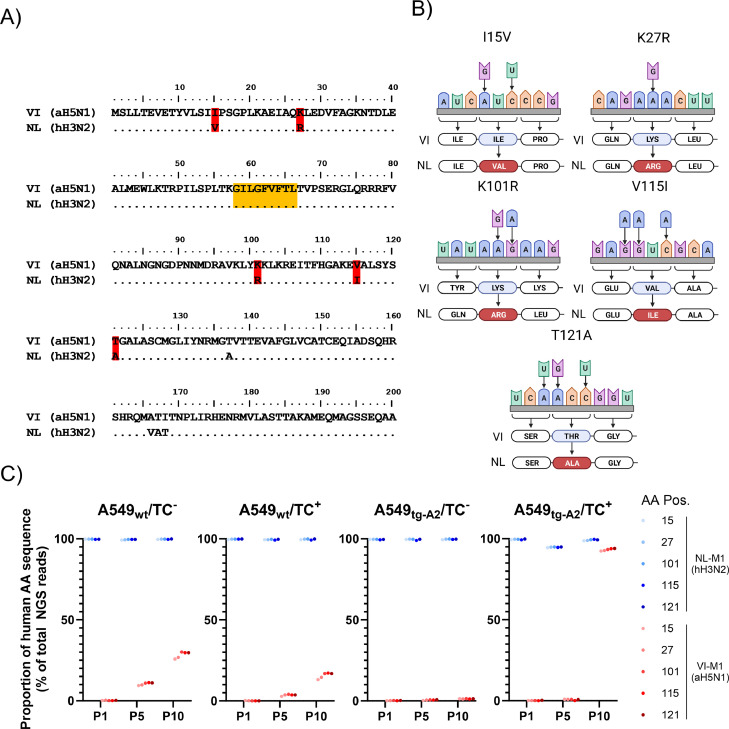
Sequence comparisons and frequency of mutations. (A) M1 sequences of hH3N2 (A/Netherlands/18/1994) and aH5N1 (A/Vietnam/1194/2004). Highlighted in yellow is the M1_58–66_ epitope. The amino acid variations of interest highlighted in red. (B) Mutations occurring at significant frequencies resulting in the depicted amino acid (AA) variations of interest (shown in A). (C) Proportion of the detected NGS reads coding for the aH5N1 AA sequence relative to the hH3N2 AA sequence at the 5 AA positions of interest. The hH3N2 virus and its progeny are indicated in blue colors, the aH5N1 virus and its progeny is indicated in red colors. The shade of the colors reflects individual AA substitutions as indicated. Depicted are the results obtained after passages 1, 5 and 10 of co-culture.

The samples obtained from passaging were subsequently prepared for NGS. Due to the limitation of read length to 600 base pairs, only the initial 600 base pairs of the M1 protein gene, which contain the coding region of the extra-epitopic positions of interest (human (M1-H3N2): Val15, Arg27, Arg101, Ile115, Ala121; avian (M1-H5N1): Ile15, Lys27, Lys101, Val115, Thr121), were amplified by PCR. Following the preparation of the NGS libraries, progeny virus samples collected after passages 1, 5, and 10, were sequenced and subjected to data analysis. Specifically, the sequences were examined for conversion at the previously mentioned positions encoding the avian-to-human amino acid substitutions I15V, K27R, K101R, V115I, T121A, and vice versa (van de Sandt et al., 2016; van de Sandt et al., 2018a) (Fig. 2A). All mutations of interest were found in both viruses passaged in A549_WT_ or A549_tg-A2_ cells, irrespective of the presence of M1_58–66_-specific T cells. A distinct pattern of codon usage was found for all five amino acid substitutions of interest, that was statistically significant (variant p-values <10^−27^) (Fig. 2B). The occurrence of all five avian-to-human mutations (Fig. 2A) were detected at significant frequencies starting from passage 5 in both A549_WT_ or A549_tg-A2_ cells. Interestingly, no other changes in nucleotide sequence that could lead to the same amino acid changes were found at a statistically significant frequency.

All five mutations relevant for T cell evasion, as well as other distinct avian-to-human mutations such as T137A and the A166V, T167A, and I168T motif (data not shown) occurred at similar rates in all samples (Fig. 1C). We observed an overall higher rate of sequence conversion, resulting in avian-to-human amino acid substitutions than vice versa (Fig. 2C). By passage 10, the average rate of changes from the avian-to-human amino acid sequences in A549_WT_ cell reached 28.4 % and 15.7 % in the absence and presence of M1_58–66_-specific T cells, respectively. Interestingly, the highest avian-to-human conversion rate (93.2 %) was observed in the sequence of progeny virus obtained at passage 10 from A549_tg-A2_ in the presence of M1_58–66_-specific T cells, while the lowest average conversion rate (1.2 %) was found in A549_tg-A2_ in the absence of M1_58–66_-specific T cells. The highest proportion of mutations from human-to-avian signature (4 %) was observed in passage 5 (A549_tg-A2_ /TC^+^), but these were not fixed and did not persist until passage 10 (Fig. 2C).

## 4 Discussion

In the present study, we tested whether M1_58–66_-specific CD8^+^ *T* cells could exert selective pressure and thus drive the accumulation of extra-epitopic amino acid substitutions associated with reduced recognition of the M1 protein of seasonal human influenza viruses by these T cells, compared to M1 of avian viruses (van de Sandt et al., 2016; van de Sandt et al., 2018a). The data from the present study confirmed that indeed, M1_58–66_-specific CD8^+^ *T* cells control replication of a virus with the M1 gene of an avian virus better than one with the M1 gene of a seasonal human influenza virus (van de Sandt et al., 2018a).

We observed a relatively low rate of accumulation of all five avian-to-human amino acid substitutions associated with reduced recognition by M1_58–66_-specific CD8^+^ *T* cells in the absence of selective pressure by these cells. However, under conditions allowing the M1_58–66_-specific CD8^+^ *T* cells to exert selective pressure, we observed levels of avian-to-human amino acid substitutions close to fixation after ten serial passages of the WSN-M-aH5N1 virus.

In order to test the selective pressure of M1_58–66_-specific CD8^+^ *T* cells *in vitro*, serial passaging of the virus in co-cultures of A549 cells and cells of an M1_58–66_-specific CD8^+^ *T* cell clone was established. Conditions were selected at which inhibitory effects on virus replication were apparent but still allowed sufficient replication of WSN-M-hH3N2 and WSN-M-aH5N1 to reach viral titers high enough for subsequent passaging. A549 cells lacking HLA-A*02:01 expression and cultures without CD8^+^ *T* cells served as negative controls. As expected and described previously (van de Sandt et al., 2018a), the inhibitory effect of M1_58–66_-specific T cells on replication of WSN-M-aH5N1 was more significant than that on WSN-M-hH3N2, confirming the relative resistant phenotype of viruses with an M1 gene of human seasonal influenza viruses.

To monitor the emergence of virus variants upon serial passaging in the absence or presence of potential selective pressure by M1_58–66_-specific T cells, the culture supernatants obtained after passages 1, 5, and 10 were subjected to Next Generation Sequencing (NGS) to detect amino acid substitutions at extra-epitopic residues 15, 27, 101, 115 and 121, known to affect recognition by M1_58–66_-specific T cells or in the epitope itself (van de Sandt et al., 2016; van de Sandt et al., 2018a). The M1_58–66_ epitope remained 100 % conserved in all analysed progeny virus preparations. This confirms the highly conserved nature of this epitope, which has been shown to be under functional constraints (Berkhoff et al., 2005; Terajima and Ennis, 2012), most likely due to overlapping with a nuclear export domain in the M1 protein (Cao et al., 2012). Mutations at the extra-epitopic residues that converted the signatures from avian-to-human, and vice versa*,* were readily detected even in passage 5, although conversion from avian-to-human signature was observed at higher frequencies. However, these amino acid substitutions were also observed in A549_wt_ cells that lack HLA-A*02:01 expression and in the absence of M1_58–66_-specific T cells.

The passaging of a molecularly cloned virus possessing an M1 gene originating from an avian virus in human lung epithelial cells, generates a quasi-species containing variants of human signature with all five amino acid substitutions of interest. Thus, the emergence of variants with substitutions at amino acid residues 15, 27, 101, 115, and 121 may not be dependent *per se* on selective pressure mediated by M1_58–66_-specific CD8^+^ *T* cells, but may also reflect adaptive changes of avian virus to mammalian host environment. Of interest, the presence of selective pressure mediated by M1_58–66_ epitope-specific T cells drove further expansion of the variants close to fixation. This observation resembles earlier findings obtained with variation in another influenza virus epitope NP_383–391_. The rapid fixation of a mutation of the HLA-B27:05 restricted epitope was explained by small selective advantages in the human population and strong bottleneck and founder effects (Gog et al., 2003). Similar processes may have been at the basis of our findings in the *in vitro* co-culture system used in the present study. IAV infection of mice, transgenic for a T cell receptor specific for the NP_366–374_ epitope, readily resulted in the emergence of virus variants with mutations in the epitope that abrogated recognition by specific T cells (Price et al., 2000). The use of HLA-A*02:01 transgenic mice would enable further investigation of the effect of the extra-epitopic residues on the M1_58–66_-specific T cell response and the emergence of virus variants with a human signature.

Interestingly, we identified specific nucleotide substitutions that led to the observed the amino acid changes (Fig. 2B). These nucleotide substitutions were the only significantly occurring changes that led to the described changes at the extra-epitopic residues. The observed consistency of the variant frequency, including those encoding the more distant amino acid substitutions (T137A and the A166V, T167A, and I168T motif), contrasts the variable nature of influenza viruses. This may be associated with secondary RNA structure involved in virus replication (Spronken et al., 2017).

Human seasonal (and pandemic) viruses that circulated since the pandemic of 1918 all had an M1 gene of human signature (van de Sandt et al., 2012). In contrast, the influenza A virus that caused the pandemic of 2009 ((H1N1)pdm09) had an M1 gene of avian signature during the first seven years of circulation in the human population. This virus did not accumulate amino acid substitutions in the extra-epitopic residues, despite selective pressure by M1_58–66_-specific T cells in the human population (van de Sandt et al., 2018b). It may take some time to acquire adaptive changes in the M1 protein and the associated immune evasive phenotype under field conditions. Investigating within-host IAV evolution may shed light in these adaptive changes (Han et al., 2021).

In the present study, we used a unique co-culture system with HLA-transgenic A549 cells, cloned T cells specific for the M1_58–66_ epitope and molecular cloned isogenic viruses. The approach enabled to investigate viral evolution under isolated selective pressure mediated by CD8^+^ *T* cells. The data suggest that the presence of amino acid residues that affect recognition of IAV by M1_58–66_-specific T cells, most likely by affecting antigen processing and epitope presentation (van de Sandt et al., 2016; Hofmann et al., 2001), is the result of host cell adaption on the one hand and selective pressure by specific T cells on the other. Continuous monitoring of the evolution of H1N1pdm09 viruses may advance our knowledge on evolution of influenza viruses with an M gene segment of avian origin.

## Funding

The Alexander von Humboldt Foundation supported this work in the framework of the Alexander von Humboldt Professorship endowed by the German Federal Ministry of Education and Research (J.M.J, A.M. G.F.R., G.S.); and the DFG-funded VIPER program (GRK 2485/1) (J.M.J). C.E.V.D.S is supported by the ARC-DECRA Fellowship (#DE200100185) and the University of Melbourne Establishment grant.

## Author statement

I certify that all authors have read and approved the final version of the manuscript, and contributed significantly to the work. The manuscript has not been published previously and is not being considered for publication elsewhere.

## CRediT authorship contribution statement

**Janina M. Jansen:** Investigation, Formal analysis, Writing – original draft. **Robert Meineke:** Writing – original draft. **Antonia Molle:** Formal analysis, Investigation. **Carolien E. van de Sandt:** Methodology, Writing – review & editing. **Giulietta Saletti:** Conceptualization, Methodology, Supervision. **Guus F. Rimmelzwaan:** Conceptualization, Writing – review & editing, Supervision, Funding acquisition.

## Declaration of competing interest

The authors declare that they have no known competing financial interests or personal relationships that could have appeared to influence the work reported in this paper.

## Data Availability

Data will be made available on request.

## Appendix Supplementary materials

Image, application 1

## References

[bib0001] Berkhoff E.G. (2004). A mutation in the HLA-B*2705-restricted NP383-391 epitope affects the human influenza A virus-specific cytotoxic T-lymphocyte response *in vitro*. J. Virol..

[bib0002] Berkhoff E.G. (2005). Functional constraints of influenza A virus epitopes limit escape from cytotoxic T lymphocytes. J. Virol..

[bib0003] Berkhoff E.G. (2007). The loss of immunodominant epitopes affects interferon-gamma production and lytic activity of the human influenza virus-specific cytotoxic T lymphocyte response *in vitro*. Clin. Exp. Immunol..

[bib0004] Boon A.C. (2002). Sequence variation in a newly identified HLA-B35-restricted epitope in the influenza A virus nucleoprotein associated with escape from cytotoxic T lymphocytes. J. Virol..

[bib0005] Cao S. (2012). A nuclear export signal in the matrix protein of Influenza A virus is required for efficient virus replication. J. Virol..

[bib0006] Gog J.R. (2003). Population dynamics of rapid fixation in cytotoxic T lymphocyte escape mutants of influenza A. Proc. Natl. Acad. Sci. USA.

[bib0007] Gras S. (2010). Cross-reactive CD8+ T-cell immunity between the pandemic H1N1-2009 and H1N1-1918 influenza A viruses. Proc. Natl. Acad. Sci. USA.

[bib0008] Han A.X. (2021). Within-host evolutionary dynamics of seasonal and pandemic human influenza A viruses in young children. eLife.

[bib0009] Hayward A.C. (2015). Natural T cell-mediated protection against seasonal and pandemic influenza. Results of the flu watch cohort study. Am. J. Respir. Crit. Care Med..

[bib0010] Hofmann M. (2001). Mechanisms of MHC class I-restricted antigen presentation. Expert Opin. Ther. Targets.

[bib0011] Jansen J.M. (2019). Influenza virus-specific CD4+ and CD8+ T cell-mediated immunity induced by infection and vaccination. J. Clin. Virol..

[bib0012] Kim H., Webster R.G., Webby R.J. (2018). Influenza virus: dealing with a drifting and shifting pathogen. Viral Immunol..

[bib0013] Koel B.F. (2013). Substitutions near the receptor binding site determine major antigenic change during influenza virus evolution. Science.

[bib0014] Krammer F. (2018). Influenza. Nat. Rev. Dis. Primers.

[bib0015] Machkovech H.M. (2015). Positive selection in CD8+ T-cell epitopes of influenza virus nucleoprotein revealed by a comparative analysis of human and swine viral lineages. J. Virol..

[bib0016] Price G.E. (2000). Viral escape by selection of cytotoxic T cell-resistant variants in influenza A virus pneumonia. J. Exp. Med..

[bib0017] Rambaut A. (2008). The genomic and epidemiological dynamics of human influenza A virus. Nature.

[bib0018] Rimmelzwaan G.F. (1998). Comparison of RNA hybridization, hemagglutination assay, titration of infectious virus and immunofluorescence as methods for monitoring influenza virus replication *in vitro*. J. Virol. Methods.

[bib0019] Rimmelzwaan G.F. (2004). Human airway epithelial cells present antigen to influenza virus-specific CD8+ CTL inefficiently after incubation with viral protein together with ISCOMATRIX. Vaccine.

[bib0020] Rimmelzwaan G.F. (2004). Sequence variation in the influenza A virus nucleoprotein associated with escape from cytotoxic T lymphocytes. Virus Res..

[bib0021] Smith D.J. (2004). Mapping the antigenic and genetic evolution of influenza virus. Science.

[bib0022] Spronken M.I. (2017). A compensatory mutagenesis study of a conserved hairpin in the M gene segment of influenza A virus shows its role in virus replication. RNA Biol..

[bib0023] Sridhar S. (2013). Cellular immune correlates of protection against symptomatic pandemic influenza. Nat. Med..

[bib0024] Terajima M., Ennis F.A. (2012). Nuclear export signal and immunodominant CD8+ T cell epitope in influenza A virus matrix protein 1. J. Virol..

[bib0025] van de Sandt C.E. (2016). Differential recognition of influenza A viruses by M158-66 epitope-specific CD8+ T cells is determined by extraepitopic amino acid residues. J. Virol..

[bib0027] van de Sandt C.E. (2018). Variation at extra-epitopic amino acid residues influences suppression of influenza virus replication by M1(58-66) epitope-specific CD8(+) T lymphocytes. J. Virol..

[bib0026] van de Sandt C.E. (2018). H1N1pdm09 influenza virus and its descendants lack extra-epitopic amino acid residues associated with reduced recognition by M158-66-specific CD8+ T Cells. J. Infect. Dis..

[bib0028] van de Sandt C.E., Kreijtz J.H., Rimmelzwaan G.F. (2012). Evasion of influenza A viruses from innate and adaptive immune responses. Viruses.

[bib0029] Voeten J.T. (2000). Introduction of the haemagglutinin transmembrane region in the influenza virus matrix protein facilitates its incorporation into ISCOM and activation of specific CD8(+) cytotoxic T lymphocytes. Vaccine.

[bib0030] Voeten J.T. (2000). Antigenic drift in the influenza A virus (H3N2) nucleoprotein and escape from recognition by cytotoxic T lymphocytes. J. Virol..

[bib0031] Woolthuis R.G. (2016). Long-term adaptation of the influenza A virus by escaping cytotoxic T-cell recognition. Sci. Rep..

